# Proteomics characterisation of central nervous system metastasis biomarkers in triple negative breast cancer

**DOI:** 10.3332/ecancer.2019.891

**Published:** 2019-01-15

**Authors:** Katerin Rojas L, Lucía Trilla-Fuertes, Angelo Gámez-Pozo, Cristina Chiva, Juan Sepúlveda, Luis Manso, Guillermo Prado-Vázquez, Andrea Zapater-Moros, Rocío López-Vacas, María Ferrer-Gómez, César Mendiola, Enrique Espinosa, Eduard Sabidó, Eva Ciruelos, Juan Ángel Fresno Vara

**Affiliations:** 1 Department of Medical Oncology, Hospital Universitario 12 de Octubre, 28041 Madrid, Spain; 2 Biomedica Molecular Medicine SL, 28049 Madrid, Spain; 3 Molecular Oncology and Pathology Lab, Instituto de Genética Médica y Molecular-INGEMM, Hospital Universitario La Paz-IdiPAZ, 28029 Madrid, Spain; 4 Proteomics Unit, Center of Genomics Regulation, Barcelona Institute of Science and Technology, 08036 Barcelona, Spain; 5 Proteomics Unit, Universitat Pompeu Fabra, 08002 Barcelona, Spain; 6 Medical Oncology Service, Hospital Universitario La Paz-IdiPAZ, 28029 Madrid, Spain; 7 CIBERONC, Instituto de Salud Carlos III, 28029 Madrid, Spain; * Katerin L Rojas and Lucía Trilla-Fuertes contributed equally to this work.

**Keywords:** breast cancer, triple negative, proteomics, central nervous system metastases, biomarkers, personalised medicine

## Abstract

**Background:**

Breast cancer (BC) is the most frequent tumour in women. Triple negative tumours (TNBC)–which are associated with minor survival rates—lack markers predictive of response to anticancer drugs. Triple negative tumours frequently metastasise to the central nervous system (CNS).

**Objective:**

The main objective of this study was to study differences in tumour protein expression between patients with CNS metastases and those without this kind of spread, and propose new biomarkers.

**Methods:**

A retrospective study was performed. Targeted proteomics and statistical analyses were used to identify possible biomarkers.

**Results:**

Proteins were quantified by a targeted proteomics approach and protein expression data were successfully obtained from 51 triple negative formalin-fixed paraffin-embedded samples. ISG15, THBS1 and AP1M1 were identified as possible biomarkers related with CNS metastasis development.

**Conclusions:**

Three possible biomarkers associated with CNS metastases in TNBC tumours were identified: ISG15, THBS1 and AP1M1. They may become markers predicting the appearance of CNS infiltration in triple negative BC.

## Introduction

Triple-negative breast cancer (TNBC) subtype represents approximately 10%–20% of all cases of breast cancer (BC) in Caucasian women and is associated with poor prognosis in terms of distant relapse-free survival and overall survival (OS) [1–3]. Patients with TNBC do not benefit from targeted therapies because a therapeutic target has not yet been established [1–3].

In murine models of BC, cyclooxygenase COX-2, an epidermal growth factor receptor (EGFR) ligand and the ST6GALNAC5 gene were identified as candidate genes related to the development of brain metastasis [4]. We have previously analysed the expression of these three genes in formalin-fixed paraffin-embedded (FFPE) samples from patients with TNBC and central nervous system (CNS) involvement and did not find such a correlation [5].

Mass-spectrometry-based proteomics is beginning to develop and to mature through a blend of enhanced instrumentation, sample preparation strategies and computational investigation [6–8]. These advances permit the distinguishing proof of thousands of proteins from tissue compatible with clinical daily practice, which is pertinent for the investigation of complex ailments. Proteomics investigations are important to describe the entire situation of flagging pathways and modified natural procedures because of the particular change set in every tumour, so protein profiling harbours the possibility of building up new patient stratifications and biomarkers advancing in individualised treatment [9].

Besides shotgun proteomics approaches, targeted proteomics procedures, such as parallel reaction monitoring (PRM) [10, 11], allow the measurement with high exactness and precision of a set of pre-selected set of proteins of interest. This technique was previously used by our group to validate predictive signatures in TNBC patients treated with adjuvant chemotherapy [8].

In this study, we aimed to identify proteins predicting CNS metastases and therapeutic target candidates in patients with TNBC. We defined three candidate biomarkers related to the presence of CNS metastasis using targeted proteomics techniques.

Brain metastases generally tend to occur late in the course of metastatic breast cancer and are typically associated with 1 and 2 year survival rates of only 20% and < 2%, respectively. In addition, several studies have reported a greater propensity of ER-negative tumours metastasizing to the brain compared with ER-positive tumors [29, 30]. Molecular detailing of subsequent events of penetration, seeding, and outgrowth in the brain is highly relevant for developing therapeutic strategies to treat or prevent, CNS metastases.

## Material and methods

### Samples

Fifty-one TNBC samples from primary tumours from I+12 Biobank (RD09/0076/00118) integrated at the Spanish Hospital Biobank Network (RetBioH; www.redbiobancos.es) were included in the study. All patients were treated with adjuvant chemotherapy. The histopathological features and tumour content of each sample were confirmed by an experienced pathologist. Eligible samples had to include at least 50% of tumour cells. Informed consent from all individual participants included in the study and approval from the Ethical Committee of Hospital Universitario La Paz (HULP PI-1417) were obtained for the conduct of the study. Samples from this cohort were analysed in previous works [8].

### Total protein extraction

Proteins were extracted from FFPE samples as described in previous works [12]. Briefly, protein extracts were prepared in 2% sodium dodecyl sulfate (SDS) following a heat-induced antigen retrieval protocol [13]. Then, protein extracts were digested with trypsin and SDS was removed using Detergent Removal Spin Columns (Pierce).

### Parallel reaction monitoring data acquisition and analysis

As described in detail previously, 37 proteins were selected for performing the PRM experiments based on their prognostic value in TNBC [8]. One to four unique peptides per protein were selected for quantification by PRM, as described in previous studies [8]. Briefly, selected peptides were spiked in the peptide mixture as isotopically labelled internal standard peptides. Each sample was analysed using an Orbitrap Fusion Lumos (Thermo Fisher Scientific) coupled to an EASY-nanoLC 1000 Ultra Performance Liquid Chromatography (UPLC) system (Thermo Fisher Scientific). A previously described scheduled PRM method was used for data acquisition [8]. Mass-spectrometry (MS) fragmentation was performed as described before [8]. The Parallel Reaction Monitoring dataset is publicly available in the Panorama web server at https://panoramaweb.org/labkey/QrYeZ2.url. Product ion chromatographic traces corresponding to the targeted precursor peptides were evaluated with Skyline software v 2.5 [8]. For each monitored peptide, a light-to-heavy ratio was calculated per patient. Ratios were transformed to the logarithmic scale (log2) and the obtained values were used as a proxy for the protein amount.

### Statistical analyses

Statistical analyses were done using GraphPad Prism v 6. Class comparison analyses were performed in BRB Array Tools developed by Dr Richard Simons. *P*-values are two-sided and *p* <0.05 are considered statistically significant.

## Results

### Patients’ characteristics

**Figure 1:**
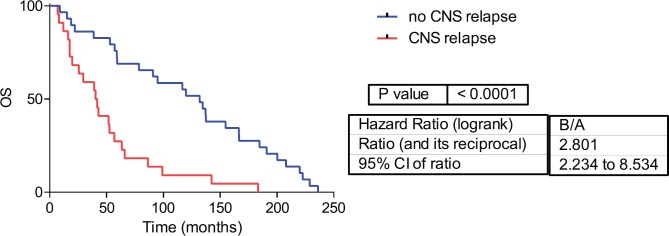
Overall survival in CNS and no CNS relapse tumours. OS= Overall survival.

**Figure 2:**
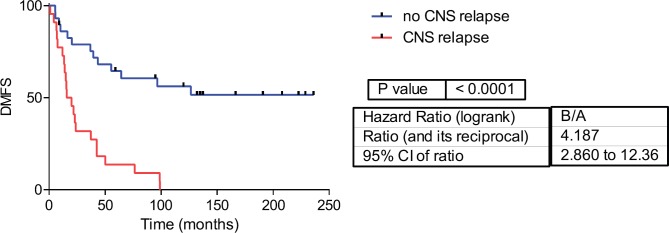
Distant metastasis-free survival in CNS and no CNS relapse tumours. DMFS= Distant metastasis-free survival.

Fifty-one samples characterised as TNBC tumours were included. Table 1 shows the patients’ clinical features. The median follow-up was 42 months (range 1–236 months). Twenty-two patients developed CNS metastases. Patients who developed CNS metastasis have a poor distant metastasis-free survival (DMFS) and OS (Figures 1 and 2).

### Targeted proteomics

Thirty-seven proteins selected for their prognostic value in TNBC tumours based on results from previous works [8] were measured using PRM targeted proteomics. One to four unique peptides per protein were chosen and these peptides were used to perform a class comparison analysis.

### Differential protein expression between CNS and no CNS TNBC metastatic tumours

**Figure 3:**
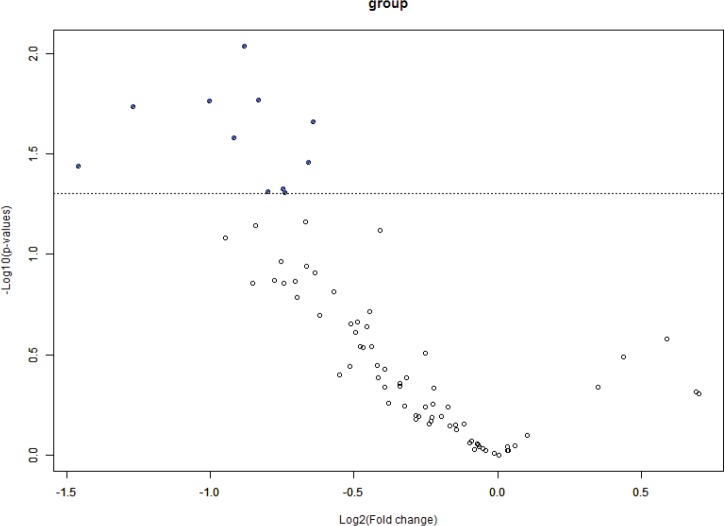
Volcano plot showing differential peptides between tumors with presence and absence of CNS metastases. Differential peptides,which are colored in blue, between CNS and no CNS metastatic tumors (p<0.05).

BRB Array Tool was employed to establish protein candidates differentially expressed between the two categories. Class comparison analysis, based on a parametric *t*-test, and volcano plot showed 11 peptides with a significant differential expression between no CNS metastasis and CNS metastasis patients (Table 2, Figure 2) (*p*-value = 0.05). However, a Mann–Whitney test (proteomics data never follow a normal distribution) reduced these 11 proteins into four candidate peptides, two of them belonging to the same protein: P05161 (interferon simulated gene 15 ubiquitin-like modifier, ISG15), Q9BXS5 (AP-1 complex subunit μ−1, AP1M1) and P07996 (thrombospondin-1, THBS1) (Figure 3). All of these proteins are overexpressed in TNBC tumours which develop CNS metastases comparing with TNBC tumours which do not develop them.

## Discussion

Proteomics technologies have been used to uncover biomarkers and molecular mechanisms associated with BC [14]. TNBC tumours frequently metastasise to the lungs and the brain [8, 12, 13]. In this work, using targeted proteomics and statistical analyses, we characterised protein expression patterns in tumours with and without CNS spread.

A group of three genes had previously been related to the presence of brain metastases in BC in murine models [4] but we failed to validate them in clinical samples [5]. Differential protein expression between the primary tumour and the brain metastasis could explain this result. We then decided to use PRM and class comparison analysis as an alternative and more powerful approach.

ISG15, an interferon (IFN)-inducible, ubiquitin-like protein, was overexpressed in TNBC tumours that developed CNS metastases. ISG15 protein is involved in numerous cellular functions, including the interferon-induced immune response and the regulation of cellular protein turnover [15, 16]. Desai *et al*. [17] demonstrated that free ISG15 and its protein conjugated form (ISGylation) are increased in human solid tumours and tumour cell lines compared with their respective normal counterparts. Other study identified in BC tumours elevated ISG15 expression when compared with normal tissue [18]. This overexpression was independent of HER2, progesterone receptor and oestrogen receptor status and correlated with an unfavourable prognosis and poor response to chemotherapy and radiation [19]. Additionally, a relationship between this protein and motility in BC tumours has been previously described [20]. ISG15 was also previously proposed as a biomarker with prognostic significance in BC; however, this study did not differentiate between BC subtypes [18].

THBS1, thrombospondin1, is an adhesive glycoprotein belonging to thrombospondins family that mediates matrix interactions and extracellular matrix structure [21]. It was overexpressed in our TNBC tumour samples which developed CNS metastases. THBS1 is an angiogenesis inhibitor [22] and it is overexpressed in BC plasma samples compared with normal samples, suggesting this molecule as a good serological marker [23]. In addition, previous studies established that THBS1 promotes metastasis in murine BC models [24]. This protein was also included in a prognostic signature for TNBC recurrence and proposed as a bad prognostic biomarker [25].

AP1M1, adaptor-related protein complex 1 μ 1 subunit, is the medium chain of the clathrin-associated protein complex AP-1. AP-1 complex is located at the Golgi and it is implicated on endocytosis [26]. It was also overexpressed in TNBC tumours with CNS metastases. It was described that AP-1 is necessary for some antigen presentation processes by major histocompatibility complex (MHC) molecules [27]. In addition, this complex was previously associated with metastasis using murine models of epidermal tumours.

Our study has some limitations. Although it is necessary to validate these findings in a new cohort, these proteins may represent novel TNBC tumour markers helpful in selecting patients who will develop CNS metastases. Although it may be interesting to correlate proteomics findings with mutational data, at this moment, Next-Generation Sequencing data from these patients are not available. They should also be explored as therapeutic targets in this clinical context. On the other hand, proteomics currently provides a powerful tool for basic research, clinical diagnostics and drug development applications in combination with advanced bioinformatics and large databases. However, an improvement in data acquisition and data analysis in targeted proteomics techniques is still necessary.

## Conclusions

Current efforts to treat CNS metastases in TNBC tumours are limited, and drugs used have proven effects on the primary tumours but lack specificity for the intracerebral tumours, passing the blood-brain barrier and intracerebral tumour cell growth. The identification of biomarkers for CNS metastases in TNBC are not well established. In this study, we proposed three new proteins related with CNS metastases in TNBC tumours. As far as we know, this is the largest cohort with CNS metastasis data analysed by proteomics. The clinical value of ISG15, AP1M1 and THBS1 as either diagnostic/prognostic factors or as therapeutic targets must be validated in independent cohorts.

## Funding

This study was funded by Instituto de Salud Carlos III, Spanish Economy and Competitiveness Ministry, Spain and by the FEDER programme, ‘Una forma de hacer Europa’ (PI12/01016, PI12/00444 and PI15/01310). LT-F is supported by the Spanish Economy and Competitiveness Ministry (DI-15-07614). GP-V is supported by Consejería de Educación, Juventud y Deporte of Comunidad de Madrid (IND2017/BMD7783). The Centre of Genomics Regulation/Universitat Pompeu Fabra Proteomics Unit is part of the ‘Plataforma de Recursos Biomoleculares y Bioinformáticos (ProteoRed)’ supported by a grant of Instituto de Salud Carlos III and Spanish Ministry of Economy and Competitiveness (PT13/0001). We acknowledge the support of the Spanish Ministry of Economy and Competitiveness, ‘Centro de Excelencia Severo Ochoa 2013–2017’ (SEV-2012-0208) and from ‘Secretaria d’Universitats i Recerca del Departament d’Economia i Coneixement de la Generalitat de Catalunya’ (2014SGR678).

## Conflicts of interest

JAFV, EE and AG-P are shareholders in Biomedica Molecular Medicine SL. LT-F is an employee of Biomedica Molecular Medicine SL. The other authors declare no competing interests.

## Ethical approval

All procedures performed in studies involving human participants were in accordance with the ethical standards of the institutional and/or national research committee and with the 1964 Helsinki declaration and its later amendments or comparable ethical standards. For this type of study, formal consent is not required.

## Figures and Tables

**Figure 4:**
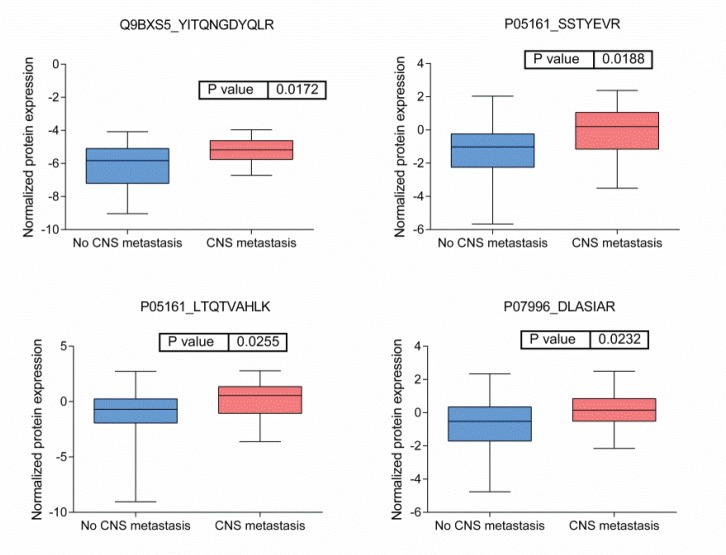
Boxplots of relative protein expression of differential peptides between tumors with no and with CNS metastases. Relative protein expression of peptides which presented a differential expression between tumors with central nervous system metastasis and tumors which no developed central nervous system metastasis.

**Table 1.** Patients’ characteristics.

Number of patients	51
Age (years)	
Median	53
Range	25–85
pT category	
pT1	8
pT2	33
pT3	6
pT4	4
pN category	
pN0	18
pN1	13
pN2	6
pN3	14
Highest G grade	
G1	1
G2	12
G3	37
Unknown	1
CNS metastasis	
Yes	22
No	29

Clinical criteria are provided according to TNM classification (http://www.cancer.gov/cancertopics/pdq/treatment/breast/healthprofessional/page3). Tumour grade is the description of a tumour based on how abnormal the tumour cells and the tumour tissue look under a microscope.

**Table 2.** Differential peptides between CNS and no CNS metastatic patients.

Parametric *p*-value	FDR	Geom mean of intensities in class 1	Geom mean of intensities in class 2	Fold change	Unique ID	Protein
0.0091723	0.348	0.014	0.026	0.54	Q9BXS5_YITQNGDYQLR	Q9BXS5
0.0170615	0.348	1.4	2.49	0.56	P53004_FGVVVVGVGR	P53004
0.0171383	0.348	0.59	1.18	0.5	P07996_DLASIAR	P07996
0.0183446	0.348	0.39	0.94	0.42	P05161_SSTYEVR	P05161
0.0218467	0.348	0.97	1.52	0.64	O75323_SGPNIYELR	O75323
0.0261312	0.348	0.066	0.12	0.53	O43747_AVEYNALFK	O43747
0.034961	0.359	0.95	1.49	0.63	Q14697_AFFAGSQR	Q14697
0.0365266	0.359	0.41	1.12	0.36	P05161_LTQTVAHLK	P05161
0.047162	0.359	2.26	3.8	0.59	Q14697_SLLLSVNAR	Q14697
0.0488091	0.359	0.014	0.025	0.57	Q5T280_TYLAGQIAR	Q5T280
0.0493982	0.359	0.84	1.4	0.6	O75323_SDMLLSR	O75323
